# Cross-Omics Analyses Reveal the Effects of Ambient PM_2.5_ Exposure on Hepatic Metabolism in Female Mice

**DOI:** 10.3390/toxics12080587

**Published:** 2024-08-13

**Authors:** Ruifeng Yan, Shaoyang Ji, Tingting Ku, Nan Sang

**Affiliations:** College of Environment and Resource, Research Center of Environment and Health, Shanxi University, Taiyuan 030006, China; yanruifeng@sxu.edu.cn (R.Y.); 15935112093@163.com (S.J.); sangnan@sxu.edu.cn (N.S.)

**Keywords:** fine particulate matter, metabolomics, transcriptomics, metabolic-associated fatty liver disease, bile acids, lipid

## Abstract

Ambient particulate matter (PM_2.5_) is a potential risk factor for metabolic damage to the liver. Epidemiological studies suggest that elevated PM_2.5_ concentrations cause changes in hepatic metabolism, but there is a lack of laboratory evidence. Here, we aimed to evaluate the effects of PM_2.5_ exposure on liver metabolism in C57BL/6j female mice (10 months old) and to explore the mechanisms underlying metabolic alterations and differential gene expressions by combining metabolomics and transcriptomics analyses. The metabolomics results showed that PM_2.5_ exposure notably affected the metabolism of amino acids and organic acids and caused hepatic lipid and bile acid accumulation. The transcriptomic analyses revealed that PM_2.5_ exposure led to a series of metabolic pathway abnormalities, including steroid biosynthesis, steroid hormone biosynthesis, primary bile acid biosynthesis, etc. Among them, the changes in the bile acid pathway might be one of the causes of liver damage in mice. In conclusion, this study clarified the changes in liver metabolism in mice caused by PM_2.5_ exposure through combined transcriptomic and metabolomic analyses, revealed that abnormal bile acid metabolism is the key regulatory mechanism leading to metabolic-associated fatty liver disease (MAFLD) in mice, and provided laboratory evidence for further clarifying the effects of PM_2.5_ on body metabolism.

## 1. Introduction

Ambient fine particulate matter (PM_2.5_) is one of the global environmental problems, and the health problems it brings have aroused widespread public concern. According to the global burden of disease (GBD) in 2021, PM_2.5_ is the leading risk factor, resulting in 8.0% of the total disability-adjusted life years (DALYs) globally [1]. In China, PM_2.5_ is responsible for the premature deaths of more than 1 million people each year [2]. Following inhalation, PM_2.5_ can cross the alveoli into the blood circulation and deposit in distal organs such as the liver, kidney, and even brain, and is thought to have serious health effects [3]. Both epidemiological and toxicological evidence suggests that PM_2.5_ may not only induce respiratory and cardiovascular diseases but also cause other adverse effects, such as metabolic disorders, liver dysfunction, and damage [4].

Metabolic-associated fatty liver disease (MAFLD) has been recognized as a hepatic feature in systemic metabolic disorders [5]. Increasing evidence suggests that fine particulate matter pollution may enhance the risk of metabolic disorders and related diseases [6,7]. An epidemiological study reveals that chronic exposure to PM_2.5_ increases the incidence of MAFLD in China [8]. Another prospective cohort study indicates that the incidence of MAFLD is 34% higher in people living in areas with high PM_2.5_ concentrations than in those with low PM_2.5_ concentrations [9]. Consistent with epidemiological evidence, toxicological studies show that exposure to air pollutants can cause liver metabolic disorders and even liver cancer [10,11]. Du et al. found that PM_2.5_ can induce hepatic steatosis in ApoE^−/−^ mice [9]. Zheng et al. reported that mice exposed to ambient PM display non-alcoholic steatohepatitis (NASH)-like phenotypes and impaired liver glucose metabolism [12]. In addition, PM_2.5_-exposed mice may develop an MAFLD phenotype, as evidenced by altered liver appearance, widespread distribution of lipid vacuoles, and balloon-like degeneration of lobular structures [13].

As a newly identified risk factor for MAFLD, PM_2.5_ has been reported to impair liver function through oxidative stress, inflammation, insulin resistance, and metabolic disorders [14]. As a key metabolic organ, the liver undergoes pathological changes in response to PM_2.5_, which may shed light on the mechanisms underlying metabolic disorders. Previous studies have found that PM_2.5_ exacerbates liver inflammation and dysregulation of lipid and sugar metabolism by activating inflammatory pathways [12]. Liu et al. confirmed that PM_2.5_ may cause fatty liver, NASH, and damaged liver sugar metabolism via hepatic IR [15]. Yan et al. found that exposure to the actual environment of PM_2.5_ can induce lipid accumulation in the liver [16]. These studies implicate the liver toxicity of PM_2.5_. However, due to the compositional complexity of PM_2.5_, it is difficult to fully understand the biological processes and toxicological mechanisms underlying adverse outcomes. The development of omics technologies provides new approaches to comprehensively understanding liver damage caused by PM_2.5_ exposure. Here, 10-month-old C57BL/6 female mice were exposed to PM_2.5_ samples collected in Taiyuan, a heavily polluted city in China. The objectives are (1) to disclose the impacts of PM_2.5_ exposure on the liver metabolic profile of mice; (2) to uncover the influences of PM_2.5_ exposure on the liver transcriptional profile of mice; and (3) to reveal the molecular mechanism of PM_2.5_ exposure resulting in lipid accumulation in the liver of mice via multi-omics analysis.

## 2. Materials and Methods

### 2.1. Collection of PM_2.5_ Samples

PM_2.5_ samples were collected at Shanxi University (Taiyuan, Shanxi Province, China) using quartz filters (Φ90 mm, Munktell, Eskilstuna, Sweden) and a TH-150CIII PM air sampler (TianHong, Wuhan, China) from November 2014 to February 2015. The contents of elements and metals within PM_2.5_ were determined using inductively coupled plasma-mass spectrometry (ICP-MS, PerkinElmer, Shelton, CT, USA). The contents of organic carbon (OC) and elemental carbon (EC) were quantified with the thermal/optical reflection (TOR) method. The contents of inorganic ions were measured via ion chromatography. Polycyclic aromatic hydrocarbons (PAHs) were determined using gas chromatography-mass spectrometry (GC-MS).

### 2.2. Animals and Exposure Experiments

C57BL/6j mice at the age of 10 months were purchased from Nanjing Junke Biological Engineering (Nanjing, China) and kept under standard conditions (24 ± 2 °C, 50 ± 5% relative humidity, and 12 h/12 h light/dark), permitted to drink and eat freely. Mice were randomly divided into a PM_2.5_-treated group and a control group. Mice in the PM_2.5_-treated group were exposed to PM_2.5_ via oropharyngeal inhalation at a dose of 3 mg/kg every other day for 4 weeks. Mice in the control group received saline prepared by sonicating blank filters in the same manner [17]. A detailed description of the experimental setup can be found in Supporting Information. At 24 h following the last exposure, mice were sacrificed to collect the livers, which were immediately stored at −80 °C.

### 2.3. LC-MS

Liver tissue (50 mg) was homogenized with 800 μL methanol (80%) and 5 μL chlorophenylalanine (2.8 mg/mL, internal standard), sonicated at 4 °C for 30 min, and maintained at −20 °C for 1 h. After centrifugation (12,000 rpm, 4 °C, 15 min), 200 μL of the supernatant was collected and commissioned to Shanghai Biotechnology Corporation for analyzed on an LC-MS platform (an UltiMate 3000 UHPLC coupled with a Q Exactive mass spectrometer, Thermo Fisher Scientific (Waltham, MA, USA)).

Firstly, the Compound Discoverer software 3.3 (Thermo Fisher Scientific, Waltham, MA, USA) was used to extract the original LC-MS data, conduct pre-processing, and then use Excel (version 16.67) to normalize and organize into a 2D data matrix, including retention time (RT), compound molecular weight (compMW), observations (samples), and peak intensity. Moreover, 3375 features in the positive ion mode (ESI^+^) and 2788 features were detected in the negative ion mode (ESI^−^) by LC-MS. The sorted data were used for multivariate statistical analysis with the SIMCA-P software 13.0 (Umetrics AB, Umea, Sweden). The principal component analysis (PCA) was adopted to evaluate the differences between the different treatment groups as well as the differences between the samples within the group. In order to further obtain information on different metabolites, we used orthogonal partial least squares discriminant analysis (OPLS-DA) to analyze the two sets of samples. DEMs were selected based on the variable importance in projection values (VIP > 1) of the OPLS-DA model and the *p*-value (*p* < 0.05) of the t-test. Then DEMs were identified by searching the Metlin online database and comparing mass-to-charge ratios or precise molecular masses of mass spectra. Metabolic pathways were analyzed using MetaboAnalyst 5.0.

### 2.4. RNA Extraction and Sequencing

RNAiso Plus Total RNA extraction reagent (TAKARA, Dalian, China) was used to extract total RNA from liver tissue. The VAHTS Stranded mRNA-seq Library Prep Kit for Illumina was used to create sequencing libraries. Seqtk was used to pre-process the original sequencing data and remove unqualified reads that might affect data quality. The gene expression levels were quantified using Stringtie to calculate the number of fragments per kilobase per million (FPKM) [18]. Then, the correlation between all samples was determined using principal component analysis. Differentially expressed genes (DEGs) between different groups were determined by edgeR [19], with *p*-value < 0.05 and fold-change ≥ 2. The function of DEG was deciphered using Gene Ontology (GO) and Kyoto Encyclopedia of Genes and Genomes (KEGG) enrichment analyses.

### 2.5. Quantitative RT-PCR

cDNA was synthesized using a reverse transcription kit (TaKaRa, Dalian, China), and RT-PCR was performed using SYBR (TaKaRa, Dalian, China) on a qTOWER 2.2 real-time PCR thermocycler (Jena, Germany). The relative expression of genes was calculated using the 2^−ΔΔCt^ method, and β-actin was used as the internal control. The primers used are shown in Table S1.

### 2.6. Data Analysis

Data were presented as the mean ± standard error (SE) and analyzed using GraphPad Prism 8 (GraphPad Software, San Diego, CA, USA). Omics data analysis and plotting were performed online (https://www.chiplot.online/index.html, URL accessed on 18 February 2023). Differences between the PM_2.5_-treated group and control groups were analyzed using a *t*-test. When *p* < 0.05, the difference was considered statistically significant.

## 3. Results

### 3.1. Chemical Compositions of PM_2.5_

**Figure 1:**
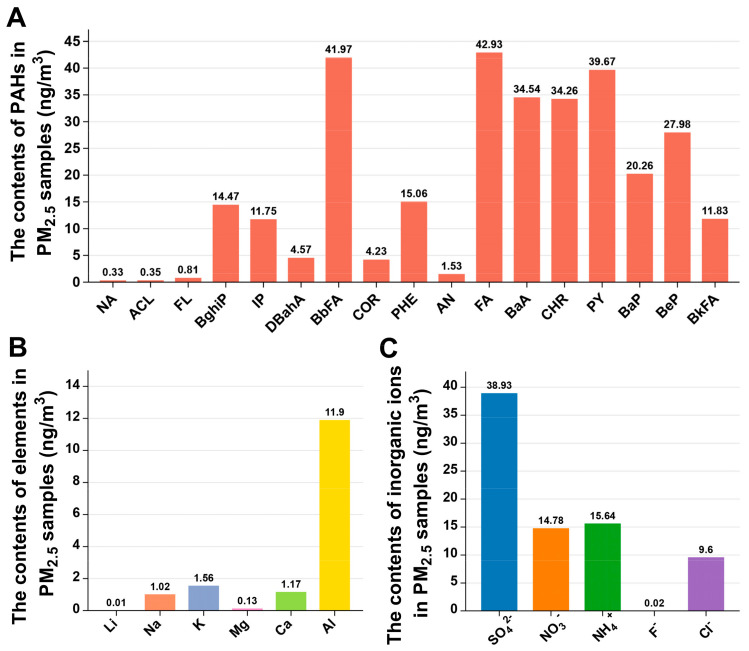
Material composition of PM_2.5_ sample. Concentrations of PAHs (**A**), elements (**B**), and inorganic ions (**C**) in PM_2.5_.

PM_2.5_ is a complex mixture of chemicals and other constituents (such as dust and microorganisms), the composition of which varies from region to region and over time due to large spatial and temporal differences in energy mix, fossil fuel combustion, and pollution treatment levels [20,21]. The toxicological essence of PM_2.5_ is the complexity of its components. Most of the previous studies have focused on the effects of PM_2.5_ mass concentrations on human health [22,23]. However, the chemical components are also the main influence of PM_2.5_ on human health. To decipher the liver toxicity induced by PM_2.5_, we first analyzed the chemical compositions of the PM_2.5_ samples used in the present study. The concentrations of PAHs, metal elements, and inorganic ions in PM_2.5_ are shown in Figure 1A. A total of 17 PAHs were detected, of which fluoranthene (FA) had the highest concentration of 42.93 ng/m^3^ (Figure 1A). There were 6 metal elements (Li, Na, K, Mg, Ca, and Al) detected in PM_2.5_, and Al was the most abundant metal element (Figure 1B). Five inorganic ions (SO_4_^2−^, NO_3_^−^, NH_4_^+^, F^−^, and Cl^−^) were detected in PM_2.5_ (Figure 1C). In addition, the concentrations of OC and EC in PM_2.5_ are 45.96 µg/m^3^ and 15.48 µg/m^3^, respectively [17].

### 3.2. PM_2.5_ Exposure Causes Changes in the Liver Metabolome

It has been reported that PM_2.5_ can enter the circulation through the alveoli and accumulate in distal organs such as the liver, kidney, and brain [3,24]. As the metabolic center of organisms, the liver is involved in many metabolic processes, such as decomposition, synthesis, transformation, and excretion [25]. Substantial evidence suggests that PM_2.5_ exposure might be relevant to metabolic liver diseases [12,26]. Studies have demonstrated that exposure to PM_2.5_ can result in hepatic insulin resistance along with fatty liver [27], non-alcoholic steatohepatitis, and compromised hepatic glucose metabolism [12,28,29]. However, the existing studies on hepatic metabolism altered by PM_2.5_ are insufficient to fully explain the mechanism of its toxicity. Therefore, we conducted a metabolomics analysis of mice exposed to PM_2.5_ to fully understand its effects on hepatic metabolism.

**Figure 2:**
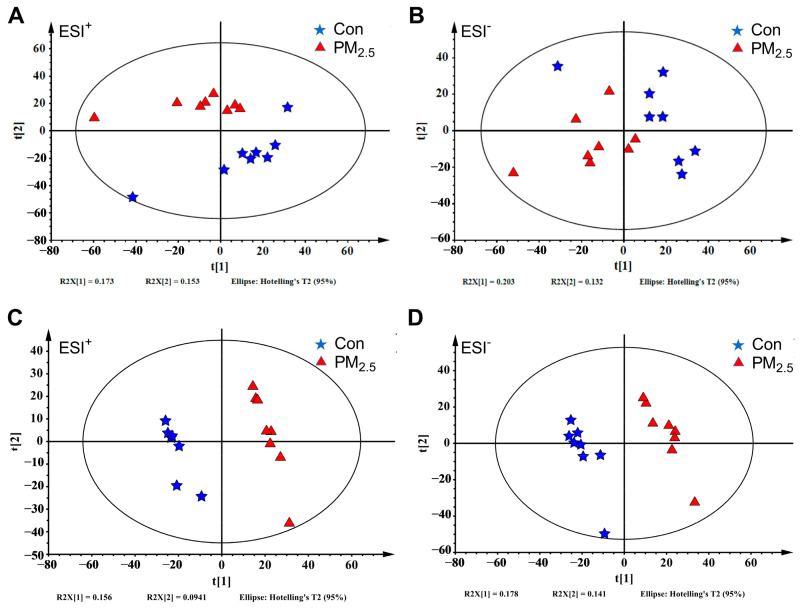
The liver metabolome changes by PM_2.5_ exposure. (**A**,**B**): The PCA analysis of the PM_2.5_ exposure group with the control group under ESI^+^ (**A**) and ESI^−^ (**B**). (**C**,**D**): The OPLS-DA plot of the PM_2.5_ exposure group with the control group under ESI^+^ (**C**) and ESI^−^ (**D**). The blue five-pointed star represents the control group, and the red triangle represents the PM_2.5_ exposure group.

We detected 3375 features by LC-MS in the ESI^+^ and 2788 features in the ESI^−^ (Table S3). PCA and OPLS-DA were applied to analyze liver metabolic profiles and visualize the overall clustering trends between PM_2.5_-exposed and control groups. As shown in Figure 2A,B, the PCA score plots showed that the PM_2.5_ treated groups were clearly separated from the control group in both ESI^+^ and ESI^−^ modes. As shown in Figure 2C,D, the OPLS-DA analysis obtained 1 principal component and 1 orthogonal component in the ESI^+^ mode (R^2^X = 0.25, R^2^Y = 0.991, Q^2^ = 0.775), as well as 1 principal component and 2 orthogonal components in the ESI^−^ mode (R^2^X = 0.379, R^2^Y = 0.998, Q^2^ = 0.807). The OPLS-DA score plot showed a significant difference, suggesting that there were significant changes in hepatic metabolites in mice after PM_2.5_ exposure.

### 3.3. Enrichment Analysis of DEMs

**Figure 3:**
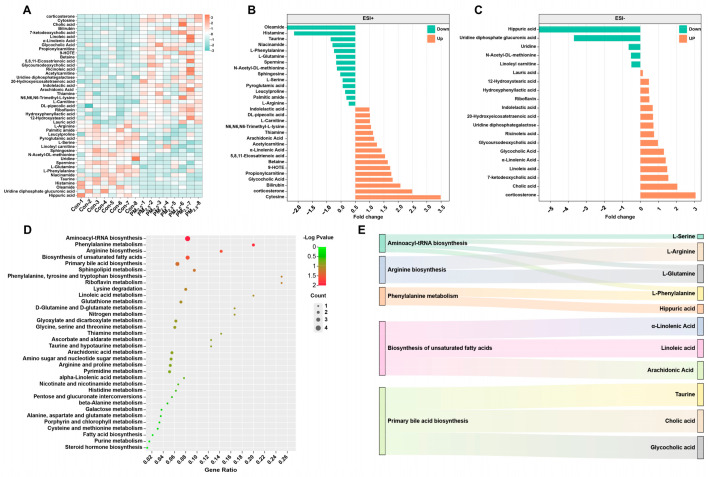
Effects of PM_2.5_ exposure on the KEGG pathway of metabolites in the liver. (**A**) The heat map shows the differential metabolites after PM_2.5_ exposure. The significantly different metabolites and their fold changes under ESI^+^ (**B**) and ESI^−^. (**C**) Green represents down-regulation, and orange represents up-regulation. (**D**,**E**) The KEGG pathway of DEM.

A total of 45 DEMs were identified in the PM_2.5_-treated mice relative to the controls (Figure 3A). In the ESI^+^ mode, 16 metabolite concentrations increased and 14 metabolite concentrations decreased (Figure 3B); in the ESI^−^ mode, 15 metabolite concentrations increased and 4 metabolite concentrations decreased (Figure 3C). The results suggest that PM_2.5_ exposure notably influences the metabolism of amino acids and organic acids. For example, PM_2.5_ exposure markedly increased the levels of organic acids (7-ketodeoxycholic acid, α-linolenic acid, linoleic acid, 5,8,11-eicosatrienoic acid, indolelactic, ricinoleic acid, 20-hydroxyeicosatetraenoic acid, and hydroxyphenyllactic acid). However, PM_2.5_ exposure also remarkably decreased the levels of amino acids (alanine, valine, serine, threonine, and ornithine).

Furthermore, we found that these DEMs belonged to 34 metabolic pathways (Figure 3D). There were five enriched metabolic pathways, including aminoacyl-tRNA biosynthesis, phenylalanine metabolism, arginine biosynthesis, biosynthesis of unsaturated fatty acids, and primary bile acid biosynthesis (Figure 3E). The results indicate that PM_2.5_ exposure mainly affects the metabolism of lipids and amino acids in the liver.

### 3.4. PM_2.5_ Exposure Causes Hepatic Transcriptomic Changes

**Figure 4:**
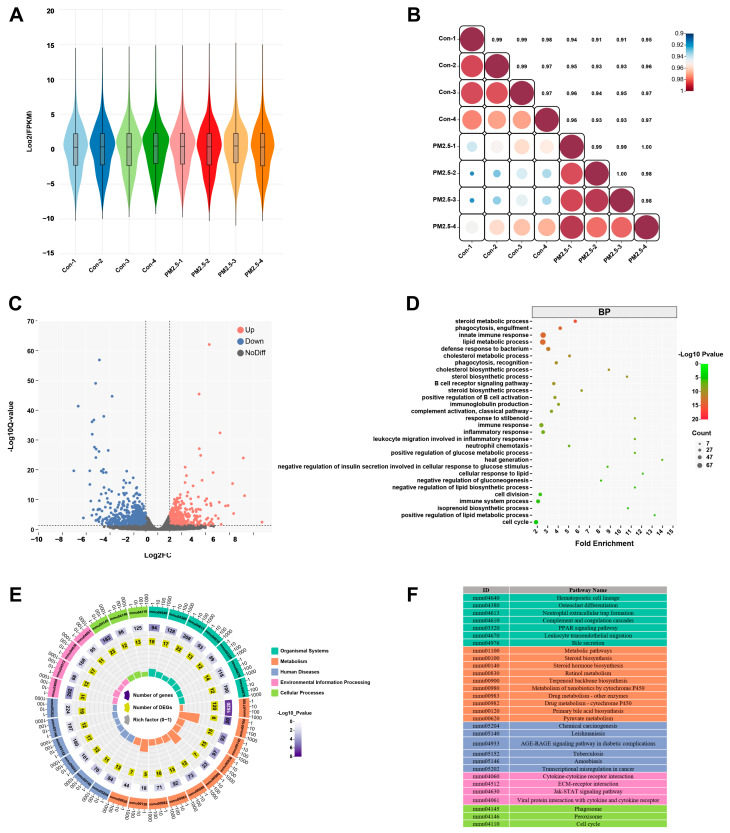
PM_2.5_ exposure causes liver transcriptomics changes. (**A**) Violin plot showing the effect of PM_2.5_ exposure on the gene expression profile of liver. (**B**) Heat map of gene expression correlation coefficient after PM_2.5_ exposure, the color shade represents the degree of correlation, and the value refers to the correlation coefficient. (**C**) Volcanic map of differentially expressed genes, red means up-regulation, blue represents down-regulation. (**D**) GO function enrichment analysis of differentially expressed genes in the PM_2.5_ exposed group compared with the control group. (**E**) The top 30 KEGG pathway enrichment scatter plot of differentially expressed genes. (**F**) The annotation of the top 30 KEGG enrichment pathways.

However, a single metabolomics analysis cannot clarify the molecular mechanism of PM_2.5_-induced liver metabolic disorders. Therefore, we observed the changes in hepatic gene expression profile caused by PM_2.5_ exposure using mRNA-seq and identified a total of 34,745 genes (Figure 4A). The gene expression-related heat map (Figure 4B) showed that the transcriptional profile of the liver was significantly different between PM_2.5_-treated and control mice. Through the analysis of transcriptomics data, we screened a total of 1081 differentially expressed genes (DEGs) (Figure 4C). Compared with control mice, there were 452 up-regulated genes and 629 down-regulated genes in PM_2.5_-treated mice. The GO enrichment results indicated that DEGs were mainly enriched in several metabolic processes (Figure 4D), including steroid metabolic process, cholesterol metabolic process, lipid metabolic process, cholesterol biosynthetic process, and sterol biosynthetic process. In addition, the KEGG pathway annotation showed that DEGs could be classified into five categories, including organismal systems, metabolism, human diseases, environmental information processing, and cellular processes (Figure 4E), and these DEGs were mainly associated with hepatic metabolism. The most significantly affected pathways included steroid biosynthesis, steroid hormone biosynthesis, retinol metabolism, terpenoid backbone biosynthesis, metabolism of xenobiotics by cytochrome P450, drug metabolism other enzymes, drug metabolism-cytochrome P450, primary bile acid biosynthesis, and pyruvate metabolism (Figure 4E,F).

### 3.5. PM_2.5_ Exposure Causes Changes in the Expression of Hepatic Bile Acid-Related Genes

**Figure 5:**
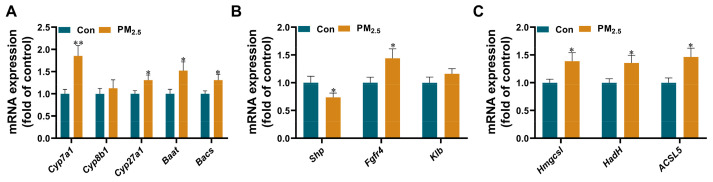
The changes of liver bile acid-related genes after PM_2.5_ exposure. The mRNA expression of bile acid synthesis (**A**), bile acid regulation (**B**), and fatty acid conversion (**C**) in the liver. The values are expressed as the mean ± SE. *n* = 8–12. * *p* < 0.05, ** *p* < 0.01. Con: control.

The results of metabolomics showed that PM_2.5_ exposure affected lipid metabolism and caused bile acid accumulation in the liver. The transcriptomics results also showed that PM_2.5_ caused abnormalities in multiple metabolic pathways, including steroid biosynthesis, steroid hormone biosynthesis, primary bile acid biosynthesis, etc. Interestingly, bile acids not only facilitate fat absorption in the intestine but also are regulators of lipid and glucose metabolism and modulate inflammation in the liver and other tissues [30]. Moreover, PM_2.5_ exposure could affect the expression of genes associated with hepatic bile acid synthesis. To prove these results, we determined the expression levels of bile acid-related genes using RT-PCR (Figure 5). The results showed that the expression of *Cyp7a1*, the rate-limiting enzyme for bile acid synthesis, in the livers of PM_2.5_-exposed mice increased by 1.85-fold compared with that of control mice, and the expression of *Cyp27a1* also increased, but the expression of *Cyp8b1* did not change. The expressions of Baat and Bacs increased by 1.52 and 1.31 times relative to the control group (Figure 5A). We also found that PM_2.5_ exposure significantly increased the expression of the fibroblast growth factor receptor 4 (*Fgfr4*) gene but not the Klothoβ (*Klb*) gene, a transmembrane protein required for *Fgfr4* activity. The expression of small heterodimer partner (*Shp*), a gene related to the regulation of hepatic bile acids, was up-regulated by 1.56 times (Figure 5B). We further tested the expression of a series of genes associated with the conversion of fatty acids to bile acids (Figure 5C). The results indicated that the expressions of *Hmgcsl*, *Hadh,* and *Acsl5* were 1.39-, 1.36-, and 1.46-fold higher in the exposure group than in the control group, respectively.

## 4. Discussion

Ambient PM_2.5_ has been considered the largest risk factor for liver damage, such as dysregulated hepatic glucose and lipid metabolism, steatohepatitis, and lipid accumulation, eventually leading to MAFLD [31,32]. As a complex mixture, PM_2.5_ may cause complicated pathological changes in the liver. However, recent studies mainly focus on the effects of PM_2.5_ exposure on specific metabolic pathways of the liver [13,28], and there are still no systematic studies on the global metabolic disorder of the liver caused by PM_2.5_ and its molecular mechanism. Omics technologies provide new ideas for the comprehensive and in-depth study of the complex toxicological effects of air pollutants on the liver. In this study, we used metabolomics and transcriptomics to investigate the hepatotoxicity of PM_2.5_.

**Figure 6:**
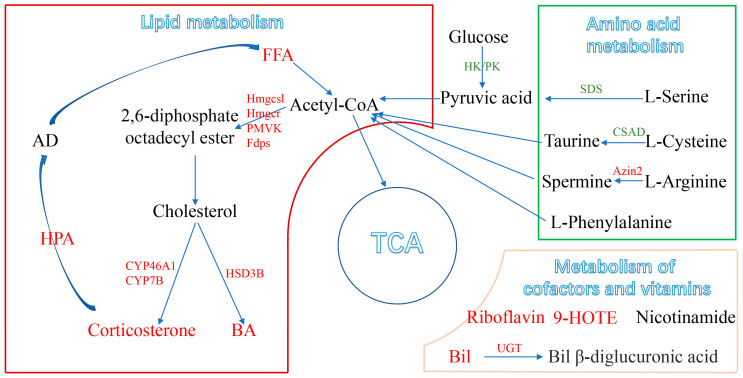
A schematic representation of disordered metabolism in the mice liver following PM_2.5_ exposure, with the relationships among metabolites, metabolic pathways, genes, and general markers. (Up-regulated metabolic pathways are marked in red and down-regulated in green). FFA: free fatty acids; BA: bile acids; TCA: tricarboxylic acid cycle; HPA: hypothalamic–pituitary–adrenal.

Based on metabolomic and transcriptomic data, we found that PM_2.5_ affected hepatic lipid metabolism in mice, increased fatty acid degradation, and enhanced the biosynthesis of cholesterols, cholesterol hormones, and bile acids, ultimately contributing to the development of MAFLD (Figure 6). Metabolomics analysis identified a total of 45 DEMs, which included increased levels of lipid metabolites and decreased levels of amino acids and their metabolites. Similar to these findings, Yan et al. also found that PM_2.5_ exposure led to elevated levels of liver triglycerides (TAGs), free fatty acids (FFAs), and cholesterol levels in female mice [33]. The results confirmed that PM_2.5_ could affect fatty acid metabolism in the liver. In addition, PM_2.5_ exposure increased the content of corticosterone in the liver. Corticosterone is a stress hormone in the glucocorticoid family that can be secreted when the hypothalamic–pituitary–adrenal (HPA) axis is activated [34]. The activation of the HPA axis can increase the secretion of adrenocortical hormones, which break down triglycerides in adipose tissue and cause the accumulation of fatty acids in the liver. Here, we found that PM_2.5_ increased the level of fatty acids in the liver, suggesting that PM_2.5_ exposure could activate the HPA axis [35]. However, Li et al. reported that PM_2.5_ inhibited the activation of HPA axis activation and reduced glucocorticoid levels [36], which is different from our result and may be attributed to the complex components of PM_2.5_ [20], the duration of exposure [37], and the different stages of disease progression [38].

Moreover, the KEGG enrichment analysis of DEMs showed that PM_2.5_ could induce hepatic lipid metabolic disorders in mice by altering four metabolic pathways, including steroid biosynthesis, primary bile acid biosynthesis, steroid hormone biosynthesis, and linoleic acid metabolism. As the substrate of the terpenoid backbone biosynthetic pathway, acetyl CoA is generated from fatty acid hydrolysis and can be converted into steroids, including cholesterol [39]. Cholesterol can synthesize bile acids as a substrate in the primary bile acid biosynthesis pathway [40]. In addition to facilitating fat absorption in the intestine, bile acids have functions in regulating glucose and lipid metabolism and modulating inflammatory responses in the liver and other tissues [30]. Abnormal accumulation of bile acids can cause toxicity, oxidative stress, and inflammation in hepatic parenchymal cells, leading to fibrosis, cirrhosis, and even end-stage liver disease [41]. Here, we found that PM_2.5_ remarkably increased the levels of cholic acid, glycocholic acid, 7-ketodeoxycholic acid, and glycoursodeoxycholic acid in the liver, indicating that PM_2.5_ could increase the conversion of cholesterol to bile acids. Consistent with this phenomenon, the KEGG enrichment analysis of both DEGs and DEMs revealed significant alterations in the primary bile acid synthesis pathway. Moreover, PM_2.5_ significantly increased the expression of core genes associated with the biosynthesis (*Cyp7a1*, *Cyp27a1*, *Baat*, and *Bacs*) and regulation (*Shp*, *Fgfr4*) of primary bile acids, suggesting that cholestasis is an important cause of PM_2.5_ hepatotoxicity.

Another finding of this study was that PM_2.5_ exerted a significant effect on hepatic amino acid metabolism in mice. PM_2.5_ also remarkably reduced the levels of arginine, phenylalanine, and other amino acids in the liver. A decrease in amino acid levels could affect the production of pyruvate, leading to the weakening of the tricarboxylic acid cycle [42]. Among them, decreased levels of arginine and spermine might inhibit glutathione metabolism, thereby reducing the formation of cysteine and taurine. Glutathione can protect red blood cells from oxidative damage and maintain iron in a reduced state, suggesting that PM_2.5_ exposure could reduce the antioxidant capacity of the liver [43]. Taurine is the major intracellular free β-amino acid and acts as a protective agent to prevent pathological changes caused by oxidative stress [44]. Glutamate is a key transamination chaperone required for the synthesis of glutathione and an essential component for protection against oxidative stress [45]. A decrease in serine levels indicates an impaired immune system in mice, leading to disease [42]. These changes caused by PM_2.5_ exposure might lead to oxidative stress and enhance hepatic amino acid catabolism, leading to a wide range of liver diseases and oxidative damage, which are consistent with swollen hepatocytes observed in histology [46]. Interestingly, PM_2.5_ significantly increased the level of betaine (a raw material for glycine synthesis) but had no effect on glycine levels. Similarly, we also found that PM_2.5_ significantly elevated the level of hippuric acid and decreased the level of serine in the liver. This phenomenon indicated that glycine combined with peptide bonds formed a large amount of hippuric acid, but hippuric acid had not been converted to serine [47]. In addition, PM_2.5_ exposure significantly reduced the contents of L-arginine, L-glutamine, L-serine, and L-phenylalanine, which might decrease the synthesis of aminoacyl-tRNA and proteins [48].

Glucose metabolism is also one of the metabolic pathways affected by PM_2.5_. PM_2.5_ can increase the level of citric acid to disturb the TCA cycle in mice, leading to imbalanced energy homeostasis [49]. Citric acid is an endogenous metabolite produced by glucose and fatty acid metabolism, which is regulated by glucose and insulin. Accumulation of citric acid decreases the activity of citrate synthase, which might affect the TCA cycle [50]. Our study showed that PM_2.5_ significantly decreased the level of uridine diphosphate glucuronic acid and increased the level of uridine diphosphate lactose, indicating that PM_2.5_ could affect glucose metabolism to a certain extent. In addition, we also observed that PM_2.5_ exposure affects the metabolism of vitamins and coenzymes, such as riboflavin, thiamine, bilirubin, and nicotinamide, in the liver. Riboflavin, thiamine, and nicotinamide are water-soluble vitamins closely related to energy metabolism and have been demonstrated to participate in fatty acid β-oxidation while affecting lipid metabolism [51]. These results also implied that PM_2.5_ impaired hepatic lipid metabolism in female mice.

## 5. Conclusions

In this study, metabolomics results revealed that PM_2.5_ exposure increased the degradation of fatty acids, promoted the biosynthesis of cholesterols, cholesterol hormones, and bile acids, and ultimately led to hepatic lipid metabolism disorder in mice. Transcriptomics results showed that the disorder of bile acid metabolism was an important reason for PM_2.5_ hepatotoxicity. In addition, PM_2.5_ also affected hepatic amino acid metabolism and glucose metabolism, ultimately leading to the development of MAFLD. In conclusion, the combined transcriptomic and metabolomic analyses comprehensively resolved the biomolecular functions and regulatory mechanisms of PM_2.5_ exposure-induced liver metabolic disorders, breaking through the deficiencies that it is difficult to identify the key pathways in a single transcriptomic analysis and impossible to explain the mechanisms in a single metabolic analysis, and providing laboratory evidence and novel insight for a comprehensive understanding of the potential hepatotoxicity of PM_2.5_.

## Data Availability

Data are contained within the article.

## Supplementary Materials

The following supporting information can be downloaded at: https://www.mdpi.com/article/10.3390/toxics12080587/s1, Table S1: DNA sequences of the primers used in quantitative real-time PCR (qPCR); Table S2: Operating parameters for ICP-MS instrument; Table S3: The detail information of 3375 features by LC-MS in the ESI+ and 2788 features in the ESI− in Appendix Excel.

## References

[1] Brauer M., Roth G.A., Aravkin A.Y., Zheng P., Abate K.H., Abate Y.H., Abbafati C., Abbasgholizadeh R., Abbasi M.A., Abbasian M. (2024). Global Burden and Strength of Evidence for 88 Risk Factors in 204 Countries and 811 Subnational Locations, 1990–2021: A Systematic Analysis for the Global Burden of Disease Study 2021. Lancet.

[2] Zhou M., Wang H., Zeng X., Yin P., Zhu J., Chen W., Li X., Wang L., Wang L., Liu Y. (2019). Mortality, Morbidity, and Risk Factors in China and Its Provinces, 1990–2017: A Systematic Analysis for the Global Burden of Disease Study 2017. Lancet.

[3] Wang H., Shen X., Liu J., Wu C., Gao J., Zhang Z., Zhang F., Ding W., Lu Z. (2019). The Effect of Exposure Time and Concentration of Airborne PM_2.5_ on Lung Injury in Mice: A Transcriptome Analysis. Redox Biol..

[4] Li D., Li Y., Li G., Zhang Y., Li J., Chen H. (2019). Fluorescent Reconstitution on Deposition of PM_2.5_ in Lung and Extrapulmonary Organs. Proc. Natl. Acad. Sci. USA.

[5] Espinosa J.S., Stryker M.P. (2012). Review Development and Plasticity of the Primary Visual Cortex. Neuron.

[6] Yang B.Y., Qian Z.M., Li S., Fan S., Chen G., Syberg K.M., Xian H., Wang S.Q., Ma H., Chen D.H. (2018). Long-Term Exposure to Ambient Air Pollution (Including PM 1) and Metabolic Syndrome: The 33 Communities Chinese Health Study (33CCHS). Environ. Res..

[7] Clementi E.A., Talusan A., Vaidyanathan S., Veerappan A., Mikhail M., Ostrofsky D., Crowley G., Kim J.S., Kwon S., Nolan A. (2019). Metabolic Syndrome and Air Pollution: A Narrative Review of Their Cardiopulmonary Effects. Toxics.

[8] Guo B., Guo Y., Nima Q., Feng Y., Wang Z., Lu R., Baimayangji, Ma Y., Zhou J., Xu H. (2022). Exposure to Air Pollution Is Associated with an Increased Risk of Metabolic Dysfunction-Associated Fatty Liver Disease. J. Hepatol..

[9] Du Z., Liang S., Li Y., Zhang J., Yu Y., Xu Q., Sun Z., Duan J. (2022). Melatonin Alleviates PM_2.5_-Induced Hepatic Steatosis and Metabolic-Associated Fatty Liver Disease in ApoE^-/-^ Mice. Oxidative Med. Cell. Longev..

[10] Jian T., Ding X., Wu Y., Ren B., Li W., Lv H., Chen J. (2018). Hepatoprotective Effect of Loquat Leaf Flavonoids in PM_2.5_-Induced Non-Alcoholic Fatty Liver Disease via Regulation of IRs-1/Akt and CYP2E1/JNK Pathways. Int. J. Mol. Sci..

[11] Ding S., Yuan C., Si B., Wang M., Da S., Bai L., Wu W. (2019). Combined Effects of Ambient Particulate Matter Exposure and a High-Fat Diet on Oxidative Stress and Steatohepatitis in Mice. PLoS ONE.

[12] Zheng Z., Xu X., Zhang X., Wang A., Zhang C., Hüttemann M., Grossman L.I., Chen L.C., Rajagopalan S., Sun Q. (2013). Exposure to Ambient Particulate Matter Induces a NASH-like Phenotype and Impairs Hepatic Glucose Metabolism in an Animal Model. J. Hepatol..

[13] Zheng Z., Zhang X., Wang J., Dandekar A., Kim H., Qiu Y., Xu X., Cui Y., Wang A., Chen L.C. (2015). Exposure to Fine Airborne Particulate Matters Induces Hepatic Fibrosis in Murine Models. J. Hepatol..

[14] Arciello M., Gori M., Maggio R., Barbaro B., Tarocchi M., Galli A., Balsano C. (2013). Environmental Pollution: A Tangible Risk for NAFLD Pathogenesis. Int. J. Mol. Sci..

[15] Liu C., Xu X., Bai Y., Wang T., Rao X., Wang A., Sun L., Ying Z., Gushchina L., Maiseyeu A. (2014). Air Pollution—Mediated Susceptibility to Inflammation and Insulin Resistance: Influence of CCR2 Pathways in Mice. Environ. Health Perspect..

[16] Yan Z., Zhang Y., Nan N., Ji S., Lan S., Qin G., Sang N. (2024). YTHDC2 Mediated RNA M6A Modification Contributes to PM_2.5_-Induced Hepatic Steatosis. J. Hazard. Mater..

[17] Ku T., Zhang Y., Ji X., Li G., Sang N. (2017). PM_2.5_-Bound Metal Metabolic Distribution and Coupled Lipid Abnormality at Different Developmental Windows. Environ. Pollut..

[18] Ma Y., Li B., Ke Y., Zhang Y., Zhang Y. (2018). Transcriptome Analysis of Rana Chensinensis Liver under Trichlorfon Stress. Ecotoxicol. Environ. Saf..

[19] Robinson M.D., McCarthy D.J., Smyth G.K. (2009). EdgeR: A Bioconductor Package for Differential Expression Analysis of Digital Gene Expression Data. Bioinformatics.

[20] Li X., Jin L., Kan H. (2019). Air Pollution: A Global Problem Needs Local Fixes. Nature.

[21] Wu D., Zheng H., Li Q., Jin L., Lyu R., Ding X., Huo Y., Zhao B., Jiang J., Chen J. (2022). Toxic Potency-Adjusted Control of Air Pollution for Solid Fuel Combustion. Nat. Energy.

[22] Liu C., Chen R., Sera F., Vicedo-Cabrera A.M., Guo Y., Tong S., Coelho M.S.Z.S., Saldiva P.H.N., Lavigne E., Matus P. (2019). Ambient Particulate Air Pollution and Daily Mortality in 652 Cities. N. Engl. J. Med..

[23] Eze I.C., Hemkens L.G., Bucher H.C., Hoffmann B., Schindler C., Künzli N., Schikowski T., Probst-Hensch N.M. (2015). Association between Ambient Air Pollution and Diabetes Mellitus in Europe and North America: Systematic Review and Meta-Analysis. Environ. Health Perspect..

[24] Li T., Hu R., Chen Z., Li Q., Huang S., Zhu Z., Zhou L. (2018). Fine Particulate Matter (PM_2.5_): The Culprit for Chronic Lung Diseases in China. Chronic Dis. Transl. Med..

[25] Huang Q., Yin P., Wang J., Chen J., Kong H., Lu X., Xu G. (2011). Method for Liver Tissue Metabolic Profiling Study and Its Application in Type 2 Diabetic Rats Based on Ultra Performance Liquid Chromatography-Mass Spectrometry. J. Chromatogr. B Anal. Technol. Biomed. Life Sci..

[26] Rajagopalan S., Park B., Palanivel R., Vinayachandran V., Deiuliis J.A., Gangwar R.S., Das L., Yin J., Choi Y., Al-Kindi S. (2020). Metabolic Effects of Air Pollution Exposure and Reversibility. J. Clin. Investig..

[27] Xu M., Ge C., Qin Y., Gu T., Lou D., Li Q., Hu L., Feng J., Huang P., Tan J. (2019). Prolonged PM_2.5_ exposure elevates risk of oxidative stress-driven nonalcoholic fatty liver disease by triggering increase of dyslipidemia. Free Radic. Biol. Med..

[28] Liu C., Xu X., Bai Y., Zhong J., Wang A., Sun L., Kong L., Ying Z. (2017). Particulate Air Pollution Mediated Effects on Insulin Resistance in Mice Are Independent of CCR2. Part. Fibre Toxicol..

[29] Sun Q., Zhang G., Chen R., Li R., Wang H. (2018). Central IKK2 Inhibition Ameliorates Air Pollution Mediated Hepatic Glucose and Lipid Metabolism Dysfunction in Mice with Type II Diabetes. Toxicol. Sci..

[30] Jiao N., Baker S.S., Chapa-rodriguez A., Liu W., Nugent C.A., Tsompana M., Mastrandrea L., Buck M.J., Baker R.D., Genco R.J. (2017). Suppressed Hepatic Bile Acid Signalling despite Elevated Production of Primary and Secondary Bile Acids in NAFLD. Gut.

[31] Reyes-Caballero H., Rao X., Sun Q., Warmoes M.O., Penghui L., Sussan T.E., Park B., Fan T.W.M., Maiseyeu A., Rajagopalan S. (2019). Air Pollution-Derived Particulate Matter Dysregulates Hepatic Krebs Cycle, Glucose and Lipid Metabolism in Mice. Sci. Rep..

[32] Zhang Z., Guo C., Chang L.Y., Bo Y., Lin C., Tam T., Hoek G., Wong M.C.S., Chan T.C., Lau A.K.H. (2019). Long-Term Exposure to Ambient Fine Particulate Matter and Liver Enzymes in Adults: A Cross-Sectional Study in Taiwan. Occup. Environ. Med..

[33] Yan Z., Li S., Chen R., Xie H., Wu M., Nan N., Xing Q., Yun Y., Qin G., Sang N. (2023). Effects of differential regional PM_2.5_ induced hepatic steatosis and underlying mechanism. Environ Pollut..

[34] Sokół R., Koziatek-Sadłowska S. (2020). Changes in the Corticosterone Level in Tooting Male Black Grouse (Tetrao Tetrix) Infected with *Eimeria* spp.. Poult. Sci..

[35] Balasubramanian P., Sirivelu M.P., Weiss K.A., Wagner J.G., Harkema J.R., Morishita M., MohanKumar P.S., MohanKumar S.M.J. (2013). Differential Effects of Inhalation Exposure to PM_2.5_ on Hypothalamic Monoamines and Corticotrophin Releasing Hormone in Lean and Obese Rats. Neurotoxicology.

[36] Li R., Sun Q., Lam S.M., Chen R., Zhu J., Gu W., Zhang L., Tian H., Zhang K., Chen L.C. (2020). Sex-Dependent Effects of Ambient PM_2.5_ Pollution on Insulin Sensitivity and Hepatic Lipid Metabolism in Mice. Part. Fibre Toxicol..

[37] Su X., Tian J., Li B., Zhou L., Kang H., Pei Z., Zhang M., Li C., Wu M., Wang Q. (2020). Ambient PM_2.5_ Caused Cardiac Dysfunction through FoxO1-Targeted Cardiac Hypertrophy and Macrophage-Activated Fibrosis in Mice. Chemosphere.

[38] Kehat I., Molkentin J.D. (2010). Molecular Pathways Underlying Cardiac Remodeling during Pathophysiological Stimulation. Circulation.

[39] Kawai H., Inabe J., Ishibashi T., Kudo N., Kawashima Y., Mitsumoto A. (2018). Short and Long Photoperiods Differentially Exacerbate Corticosterone-Induced Physical and Psychological Symptoms in Mice. Biomed. Res..

[40] Jones H., Alpini G., Francis H. (2015). Bile Acid Signaling and Biliary Functions. Acta Pharm. Sin. B.

[41] Pan P.H., Wang Y.Y., Lin S.Y., Liao S.L., Chen Y.F., Huang W.C., Chen C.J., Chen W.Y. (2022). 18β-Glycyrrhetinic Acid Protects against Cholestatic Liver Injury in Bile Duct-Ligated Rats. Antioxidants.

[42] Shi C., Han X., Mao X., Fan C., Jin M. (2019). Metabolic Profiling of Liver Tissues in Mice after Instillation of Fine Particulate Matter. Sci. Total Environ..

[43] Ribeiro J.d.P., Kalb A.C., Campos P.P., La Cruz A.R.H.D., Martinez P.E., Gioda A., de Souza M.M., Gioda C.R. (2016). Toxicological Effects of Particulate Matter (PM_2.5_) on Rats: Bioaccumulation, Antioxidant Alterations, Lipid Damage, and ABC Transporter Activity. Chemosphere.

[44] Aydın A.F., Çoban J., Doğan-Ekici I., Betül-Kalaz E., Doğru-Abbasoğlu S., Uysal M. (2016). Carnosine and Taurine Treatments Diminished Brain Oxidative Stress and Apoptosis in D-Galactose Aging Model. Metab. Brain Dis..

[45] Larsson T., Koppang E.O., Espe M., Terjesen B.F., Krasnov A., Moreno H.M., Rørvik K.A., Thomassen M., Mørkøre T. (2014). Fillet Quality and Health of Atlantic Salmon (*Salmo salar* L.) Fed a Diet Supplemented with Glutamate. Aquaculture.

[46] Turner M.C., Krewski D., Pope C.A., Chen Y., Gapstur S.M., Thun M.J. (2011). Long-Term Ambient Fine Particulate Matter Air Pollution and Lung Cancer in a Large Cohort of Never-Smokers. Am. J. Respir. Crit. Care Med..

[47] Gatley S.J., Sherratt H.S.A. (1977). The Synthesis of Hippurate from Benzoate and Glycine by Rat Liver Mitochondria. Submitochondrial Localization and Kinetics. Biochem. J..

[48] Nagel G.M., Doolittle R.F. (1991). Evolution and Relatedness in Two Aminoacyl-TRNA Synthetase Families. Proc. Natl. Acad. Sci. USA.

[49] Song Y.N., Dong S., Wei B., Liu P., Zhang Y.Y., Su S.B. (2017). Metabolomic Mechanisms of Gypenoside against Liver Fibrosis in Rats: An Integrative Analysis of Proteomics and Metabolomics Data. PLoS ONE.

[50] Cui Y., Han J., Ren J., Chen H., Xu B., Song N., Li H., Liang A., Shen G. (2019). Untargeted LC-MS-Based Metabonomics Revealed That Aristolochic Acid I Induces Testicular Toxicity by Inhibiting Amino Acids Metabolism, Glucose Metabolism, β-Oxidation of Fatty Acids and the TCA Cycle in Male Mice. Toxicol. Appl. Pharmacol..

[51] Bian X., Gao W., Wang Y., Yao Z., Xu Q., Guo C., Li B. (2019). Riboflavin Deficiency Affects Lipid Metabolism Partly by Reducing Apolipoprotein B100 Synthesis in Rats. J. Nutr. Biochem..

