# Supplementary material for: Cross-Omics Analyses Reveal the Effects of Ambient PM2.5 Exposure on Hepatic Metabolism in Female Mice
Source: Toxics. 2024 Aug 13;12(8):587. doi: 10.3390/toxics12080587 (PMC11360593; doi:10.3390/toxics12080587)
Supplement: Supplementary file 1 [file toxics-12-00587-s001.zip › toxics-3107330-supplementary.pdf]

Supporting Information for

Cross-omics analyses reveal the effects of ambient $PM_{2.5}$ expo-sure on hepatic metabolism in female mice

Ruifeng Yan[1], Shaoyang Ji[1], Tingting Ku* and Nan Sang

[1]R.Y. and S.J. contributed equally to this work

* Corresponding author: Tingting Ku

This PDF file includes:

Supporting text
Tables S1 to S2

Other supporting materials for this manuscript include the following:

Appendix Excel.

Contents

Text S1. Materials and Methods

Table S1. DNA sequences of the primers used in quantitative real-time PCR (qPCR)**.**

Table S2. Operating parameters for ICP-MS instrument.

Table S3. The detail information of 3375 features by LC-MS in the ESI^+ and 2788 features in the ESI^- in Appendix Excel.

Text S1.

Experimental setup

A total of 40 mice were randomly divided into the Con and $PM_{2.5}$ groups (20 mice per group). After 1 weeks of exposure, the animals were allocated for different assays, with 8 mice from each group for metabolomics analyses, 3 for transcriptomics sequencing analysis, and 9 for PCR assays. During the exposure, $PM_{2.5}$-treatment mice were treated with 3 mg/kg body weight of $PM_{2.5}$ by oropharyngeal aspiration every other day for 4 weeks. The administered dose was 1 mL of stock solution (3 mg/mL) per gram of mouse body weight, and the final dose reached 3 mg/kg bw. Animals in the control group were treated with saline that was processed with ultrasonic vibration of a blank membrane filter.

Detection of the elements and metals content

The contents of elements in the live were examined using ICP-MS (PerkinElmer, Boston, USA). Briefly, the samples were quantified and digested thoroughly at 180°C for 20 min with microwave-assisted digestion (Yiyao Instrument Technology Development Co., Ltd.,Shanghai, China). After cooling, the residues were transferred to 50-mL flasks and diluted with dilute nitric acid (1%). The metal contents were eventually determined by ICP-MS. The working conditions and instrument parameters are listed in Table S2.

Detection of the OC and EC content

The contents of organic carbon (OC) and elemental carbon (EC) were quantified with the thermal/optical reflection (TOR) method. Firstly, a programmed temperature rise is conducted in an inert environment to analyze the contents of the four carbon components, namely OC1, OC2, OC3, and OC4. Then, in an oxygen-containing environment, the programmed temperature rise proceeds to analyze the contents of the three carbon components, namely EC1, EC2, and EC3. A 633 nm He-Ne laser is employed to quantify the OP formed during the carbonization process of organic carbon, and the concentrations of OC and EC in $PM_{2.5}$ samples are calculated. Two parallel samples are selected for the entire process quality control of sampling and analysis. In this study, the concentrations of OC and EC in blank samples are both less than 5% of those in the actual samples.

Detection of the PAHs content

PAHs quantification was performed on a gas chromatograph (GC) (8890; Agilent, USA) equipped with a mass spectrometer (MS) (7000D; Agilent, USA). The GC separation was accomplished using a DB-5MS capillary column (30 m × 0.25 mm × 0.25 μm film thickness, Agilent). Helium (99.999% purity) served as the carrier gas at a flow rate of 1.0 mL/min. Splitless injections of 1 μL of the sample extracts were performed at a temperature of 260 °C. The oven temperature program initiated at 60 °C, ramped up to 180 °C at a rate of 25 °C/min (held for 1 min), then to 280 °C at a rate of 3 °C/min (held for 10 min). The MS operated in electron ionization (EI) and multiple reaction monitoring (MRM) modes. The ion source, transfer line, and quadrupole temperatures were set at 300 °C, 280 °C, and 200 °C, respectively, and electron energy was set at 70 eV.

Table S1. DNA sequences of the primers used in quantitative real-time PCR (qPCR).

Gene	*Primer*	*Sequence (5′-3′)*
Cyp7a1	*Cyp7a1-F*	*ACTTCTGCGAAGGCATTTGG*
	Cyp7a1-R	*GCATCTCCCTGGAGGGTTTT*
Cyp8b1	*Cyp8b1-F*	*CTCCCCATAAGACGCCATCC*
	Cyp8b1-R	*TGAGTCAAGTGTGGGTGAGC*
Cyp27a1	*Cyp27a1-F*	*GGCCCTGTACCACCTTTCAA*
	Cyp27a1-R	*GCGCAGGGTCTCCTTAATCA*
Baat	*Baat-F*	*GCTGTTCCTCTCAGTGCTCTT*
	Baat-R	*CTCCAAGTGAGGGGTCATGC*
Bacs	*Bacs-F*	*TGTAACGTCCCTGAGCAACC*
	Bacs-R	*TAAGCCCACATTGCCCTCTG*
Shp	*Shp-F*	*CCAGGGAAATAACCCAGATCCA*
	Shp-R	*AGGTCAGCATAGGTGAGGGT*
Fgfr4	*Fgfr4-F*	*AGGTGGTCAGTGGGAAGTCTG*
	Fgfr4-R	*CTGCTCCAGGATTGGGGCTA*
Klb	*Klb-F*	*TCCAGACCTTTGGAGACCGT*
	Klb-R	*TGATCAGGTTGTGTCCCACAG*
Acsl5	*Acsl5-F*	*GTCAGAAAGCCCGGCTATGG*
	Acsl5-R	*CAACAGGAAATTCAGACCCTGG*
Hmgcsl	*Hmgcsl-F*	*ATTCAGGTGAGCACCTCCTCC*
	Hmgcsl-F	*GAGTGGTCAGCCATCTGTGG*
Hadh	*Hadh-F*	*CAACCCCGTGCCTATGATGA*
	Hadh-R	*GTATGGCACCAAGAGTCGGT*
β-Actin	*β-Actin -F*	*GCTTCTTTGCAGCTCCTTCGT*
	β-Actin -R	*ATATCGTCATCCATGGCGAAC*

Table S2. Operating parameters for ICP-MS instrument.

Parameters	Setting	Parameters	Setting
Radio-frequency power (W)	1050	Analog voltage (V)	-1900
Flow rate of atomizing gas (L/min)	0.86	Impulse voltage (V)	900
Flow rate of sample uptake (L/min)	1.2	Scanning mode	Jump peak
Ion lens voltage (V)	8.25	Residence time (ms)	50
Flow rate of coolant gas (L/min)	13.8	Times of repetition	3
Flow rate of carrier gas (L/min)	0.98	Integration time (ms)	500
